# Computing the statistical significance of optimized communities in networks

**DOI:** 10.1038/s41598-019-54708-8

**Published:** 2019-12-05

**Authors:** John Palowitch

**Affiliations:** Google Research, San Francisco, 94105 USA

**Keywords:** Statistics, Computer science

## Abstract

In scientific problems involving systems that can be modeled as a network (or “graph”), it is often of interest to find network *communities* - strongly connected node subsets - for unsupervised learning, feature discovery, anomaly detection, or scientific study. The vast majority of community detection methods proceed via optimization of a quality function, which is possible even on random networks without communities. Therefore there is usually not an easy way to tell if a community is “significant”, in this context meaning more internally connected than would be expected under a random graph model without communities. This paper generalizes existing null models and statistical tests for this purpose to bipartite graphs, and introduces a new significance scoring algorithm called Fast Optimized Community Significance (FOCS) that is highly scalable and agnostic to the type of graph. Compared with existing methods on unipartite graphs, FOCS is more numerically stable and better balances the trade-off between detection power and false positives. On a large-scale bipartite graph derived from the Internet Movie Database (IMDB), the significance scores provided by FOCS correlate strongly with meaningful actor/director collaborations on serial cinematic projects.

## Introduction

Many natural systems can be modeled as a network, with network nodes representing entities and network edges representing links or relationships between those entities. As such, a wide variety of network models and graph algorithms have been developed, generalized, and improved over many decades, forming the field of network science and the study of complex networks^1^. A sub-field of network science is focused on methodology for and applications of “community” detection. Defined loosely, a community is a subset of nodes in a network that are more connected to each other than they are to other nodes. There are many distinct, precise definitions of a community, with utilities that vary by application^2^. In practice, the purpose of community detection is to discover dynamics or features of the networked system that were not known in advance. Community detection has been profitably applied to naturally arising networks in diverse fields like machine learning, social science, and computational biology^3^.

Usually, community detection proceeds by finding a division or cover of the network that is optimal with respect to some quality function or search procedure. Often, a *partition* of the network is the object being optimized with the quality function. Arguably the most commonly used and studied quality function for partition optimization is modularity, which is the sum of the first-order deviations of each community’s internal edge count from a random graph null model^4^. Other community detection methods aim to find a *collection* of communities, where the requirement that communities be disjoint and exhaustive is relaxed (partitions are also collections). In some approaches, collections of communities are found by optimizing communities one-by-one, according to a community-level quality function^5–7^.

Hundreds of distinct community detection methods have been introduced in recent decades. Despite this, relatively few articles discuss issues of statistical significance related to community detection. In particular, there is often no immediate way to determine if the communities returned by a community detection algorithm are of higher “quality” than would be expected (on average) if the algorithm were run repeatedly on a random graph model without true communities. When significance is discussed or addressed, it is usually with reference to the overall partition, rather than individual communities^8,9^.

This paper introduces a method called Fast Optimized Community Significance (FOCS) for scoring the statistical significance of individual communities that, importantly, have been optimized by a separate method. As discussed in the Methods section, there are several approaches for scoring optimized communities. This paper makes two advancements in this area:

1. Null models for scoring optimized communities are made explicit and newly generalized to bipartite graphs.
2. A new method (FOCS) is introduced which enjoys some benefits over existing methods:
  A core algorithm that is transparent, easy to implement, and applies freely to either unipartite or bipartite graphs.
  Higher numerical stability and 10–100x faster runtimes.
  Type-I error control with improved power to detect ground-truth communities.

For the rest of this introduction, we turn to a discussion of related work. First, we should note that there are community detection and clustering methods which use significance tests to *discover* communities^6,7^, and some methods for assessing the significance of community partitions as a whole [e.g.^8–10^]. However, none of these provide significance tests for individual communities, and are therefore out-of-scope in this work.

Recently^11^, proposed a simulation-based method called the QS-Test for inference on individual communities via community quality scores. The QS-Test generates a large sample of null networks with the configuration model^12^, each with a degree distribution matching the observed network. On each null network, a community detection algorithm is run, and a kernel density estimate of the quality score’s null distribution is computed from the resulting communities. For inference, observed quality scores from the original network are compared against the estimated null. The QS-Test approach is quite general, as it can be applied with many quality scores, and any community detection algorithm. Furthermore, it is (at least in principle) evaluating community significance against a direct estimate of its quality function’s null distribution. However, a drawback to the approach is that it is not scalable to large networks, as it requires many independent simulations of networks that have the same amount of edges as the original network, as well as a community detection run on each simulated network.

Another simulation-based method introduced in^13^ uses parameteric bootstrapping of the *observed* network, and a measure of cluster stability across the bootstrapped networks, to assess “significance” of individual clusters. This approach does not measure *statistical* significance, as it is based on cluster persistence across bootstrap samples rather than a null model. In this work we only consider methods that compute statistical significance of clusters.

An approach introduced in^14^ uses an analytical approximation to compute the statistical significance of a community. The authors of ^14^ first define a null model for simple undirected graphs. The authors reason that, if the community is a false positive, the community in-degree of its *worst*-connected node should be distributed as the maximum order statistic of the external nodes, with respect to the null model. They derive a basic significance score from this observation, and then propose a modified version of the score for an *optimized* community. The particulars of this method will be discussed further in the Methods section, as the FOCS approach has a similar foundation. Building upon their score based on a community’s worst node, the authors then propose to test nodes up to the *k*-th worst node in the community. They show through empirical studies that this “B-Score” (for “border” score) is more powerful while controlling false-positive rates in null networks. The strengths of the B-Score approach over the QS-Test is that it is analytical and therefore can be faster to compute on a single machine. A drawback of the approach is that it contains more approximations to the null distribution than the QS-Test, and does not have the notion of effect-size or quality score which is inherent to that method.

## Methods

In this paper, a network is denoted by $G\,:=(V,A)$, where *V* is a set of vertices and *A* is an adjacency matrix. Let the operator |·| denote the size of a set, and let $n\,:\,=|V|$. For *u*, $v\in V$, the entry $A(u,v)$ is equal to the number of edges between nodes *u* to *v*. We consider only *undirected* networks for which $A(u,v)=A(v,u)$. Denote the degree of $u\in V$ by ${d}_{u}\,:\,={\sum }_{v\in V}\,A(u,v)$. Let $C\subseteq V$ denote a community or any node subset. With a slight abuse of notation, let ${d}_{C}\,:\,={\sum }_{u\in C}\,d(u)$ be the total degree of a subset. Analogously, ${d}_{u}(C)\,:\,={\sum }_{v\in C}\,A(u,v)$, and ${d}_{C}(C^{\prime} )\,:\,={\sum }_{u\in C}\,{d}_{u}(C^{\prime} )$, where $C^{\prime} \,:\,=V\backslash C$. Note that for undirected networks, ${d}_{C}(C^{\prime} )={d}_{C^{\prime} }(C)$ for any $C\subseteq V$.

### Fast Optimized-Community Significance (FOCS)

Given a community of interest *C*, the focus of the core FOCS algorithm is on the edge distribution of external nodes $u\in C^{\prime}$, under a random graph null model. The null model is a community-conditional version of the standard configuration model^12^, described here first for unipartite graphs, and then newly extended to bipartite graphs later in this section. The null model breaks edges coming out of *C*, and all edges internal to *C*′, and randomly re-assigns the edges of *u* without replacement. This process models, in a sense, the typical “exclusivity” of *C* expected in a graph with the same degree distribution but with a uniformly random edge distribution. Under the model, the degree of *u* in *C* has a hypergeometric probability mass function:

1 $$P({d}_{u}(C)=a)=\frac{(\begin{array}{c}{d}_{C}(C^{\prime} )\\ a\end{array})\,(\begin{array}{c}{d}_{C^{\prime} }(C^{\prime} )\\ {d}_{u}-a\end{array})}{(\begin{array}{c}{d}_{C^{\prime} }\\ {d}_{u}\end{array})}.$$

The null model in^14^ is similar and also involves the hypergeometric distribution, but with different parameters. If *C* is optimized, the least-connected or “worst” in-community node $w\in C$ should be at the maximum quantile of *P*, among external nodes. Therefore, we measure the statistical significance of *C* by testing the quantile of the worst node, via p-values. Because standard p-values for discrete distributions are not exact, we now introduce an efficient simulation-based method for deriving exact Uniform p-values from a discrete variable.

#### Continuity correction for in-degree quantiles

Define the cumulative distribution function of *P* as $g(a;u,C):\,=P({\tilde{d}}_{u}(C)\le a)$, where ${\tilde{d}}_{u}(C)$ is the random version of ${d}_{u}(C)$ with respect to *P*. Note that $g(\cdot ;u,C)$ is not continuous and therefore ${g}_{P}({\tilde{d}}_{u}(C);u,C)$ is not a uniform random variable. We use a stochastic approach to construct a uniform CDF of *P*, from which to derive p-values for the FOCS algorithm. Denote a uniform variable on the range $[{x}_{1},{x}_{2}]$ by $U[{x}_{1},{x}_{2}]$, and define

2 $$\hat{g}(a;u,C):\,=U[g(a-1;u,C),g(a;u,C)].$$

The following theorem shows that $\hat{g}({\tilde{d}}_{u}(C);u,C)$ is a uniform random variable with respect to *P*.

##### **Theorem 1:**

*Let* $\tilde{d}$ *be a discrete random variable on a finite set* $S\subset {\mathbb{R}}$. *Let* $g:S\mapsto [0,1]$ *be its cumulative distribution function defined* $g(a)\,:\,=P(\tilde{d}\le a)$. *Order the elements of S so that for* ${a}_{i},{a}_{j}\in S$, $g({a}_{i})\le g({a}_{j})$ *if and only if* $i\le j$. *By convention*, *define* ${a}_{0}\,:\,={a}_{1}-1$. *Define the uniform random variable* $\hat{g}(a)\,:\,=U[g(a^{\prime} ),g(a)]$ *where* $a^{\prime} ={a}_{i-1}$ *when* $a={a}_{i}$. *Then for any* $x\in [0,1]$, *the conditional random variable* $\hat{g}(\tilde{d})$ *satisfies* $P(\hat{g}(\tilde{d})\le x)=x$.

*Proof*. First write the distribution of $\hat{g}(\tilde{d})$ as the sum

3 $$P(\hat{g}(\tilde{d})\le x)=\sum _{a\in S}\,P(\{\tilde{d}=a\}\cap \{\hat{g}(\tilde{d}) < x\})=\sum _{a\in S}\,P(\tilde{d}=a)P(\hat{g}(a)\le x)$$

Define $\Delta (a):\,=g(a)-g(a^{\prime} )$. By definition of $\hat{g}$, accounting for *x* with respect to the range $[g(a^{\prime} ),g(a)]$, we have

4 $$P(\hat{g}(a)\le x)={\int }_{g(a^{\prime} )}^{{\rm{\min }}(x,g(a))}\,\Delta {(a)}^{-1}1(g(a^{\prime} )\le x)dz$$

Note that $\Delta (a)=P(\tilde{d}=a)$, by definition of *g*. Therefore,

5 $$P(\tilde{d}=a)P(\hat{g}(a)\le x)=\Delta (a)\,{\int }_{g(a^{\prime} )}^{{\rm{\min }}(x,g(a))}\Delta {(a)}^{-1}1(g(a^{\prime} )\le x)dz={\int }_{g(a^{\prime} )}^{{\rm{\min }}(x,g(a))}\,1(g(a^{\prime} )\le x)dz.$$

Combining Eqs. 3 and 5, we have $P(\hat{g}(\tilde{d})\le x)={\sum }_{a\in S}\,{\int }_{g(a^{\prime} )}^{{\rm{\min }}(x,g(a))}\,1(g(a^{\prime} )\le x)dz={\int }_{0}^{x}\,dz$, which completes the proof.

Due to the uniformity of $\hat{g}({\tilde{d}}_{u}(C);u,C)$ as established by Theorem 1, we define a node-wise p-value function $p(u,C):\,=1-\hat{g}({d}_{u}(C);u,C)$ to find the worst community node. Order the nodes in *C* as ${u}_{1},{u}_{2},\ldots ,{u}_{|C|}$ such that $p({u}_{1},C)\ge p({u}_{2},C)\ge \ldots \ge p({u}_{|C|},C)$. With this ordering, we call *u*_1_ the “worst” node as its in-degree ${d}_{{u}_{1}}(C)$ falls the lowest against the null distribution *P*. Thus, by Theorem 1, it is possible to test the significance of *C* by comparing $p({u}_{1},C)$ to the distribution of the minimum of $|C^{\prime} |+1$ uniform random variables from the range $[p({u}_{2},C),1]$. Writing as ${F}_{(1)}(x;{u}_{2},C,m)$ the cumulative distribution function of the minimum of *m* uniform order statistics on that range, the significance score on which FOCS is based is defined

6 $$f(C)={F}_{(1)}(p({u}_{1},C);{u}_{2},C,|C^{\prime} |+1).$$

The score *f*(*C*) has the standard interpretation given to traditional p-values - a low value of *f*(*C*) implies that the connectivity observed in *C* is unlikely to have arisen in a random (community-less) network.

The idea of using the worst node of a community to test optimized communities was introduced in^14^. However, those authors proposed adjusted hypergeometric parameters that account for perfect community optimization, which is more fully described in their publication. The approach in the present paper relies on the assumption that in practice, communities are rarely perfectly optimized. In fact, exact modularity optimization is exponentially complex and computationally infeasible on networks with any more than a few hundred nodes^15^. Furthermore, the modularity maximization surface is glassy, with many local optima extremely close to the true maximum^16^. This suggests that for a locally optimized false-positive community, the distribution of worst nodes can be adequately described by the simpler model outlined above, despite the optimization. Note that the null model described is well-defined on graphs that allow for multiple edges between nodes.

There may be multiple nodes in an optimized community that are spurious, in the sense that moving them to another community would not significantly change the quality score of the overall partition^16^. Therefore, instead of a single worst node, a “worst set” of nodes may be a more robust test subject for determining significance. To test a worst set of nodes, the FOCS method computes *f*(*C*), removes the worst node, re-computes *f*, and so-on until a given proportion $\rho$ nodes are tested. The minimum *f*(*C*) observed over the iterations is used as the FOCS score. We found that the globally-applied setting of $\rho =0.25$ worked well on simulations and data analyses, and nearby values performed similarly. This is intuitive, as testing the “best” or most interior nodes of an optimized community ($\rho  > 0.5$) may lead to spuriously low values of *f*, even under the null, since the community has been optimized.

The complete FOCS algorithm, as just described, is given in Algorithm 1. The algorithm has multiple practical benefits. First, it is simple to implement and fast to compute. Second, testing multiple worst-nodes is beneficial when there are ground-truth communities in the network. As mentioned above, modularity optimization is necessarily local, and thus even real communities may be contaminated with noise nodes. The FOCS algorithm is able to bypass noise nodes in a real community, increasing detection power. Note that removal of the worst node changes the index of the second-worst node *u*_2_, and therefore the FOCS significance score naturally accounts for previously computed scores. The B-Score approach employs a similar accounting via conditional probabilities. Neither method uses multiple-testing across significance scores. The benefits and trade-offs of multiple testing in this setting is an area for future research.

**Algorithm 1:**
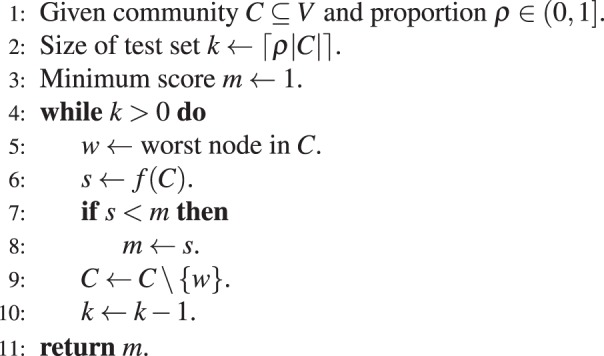
FOCS.

#### Extension to bipartite and directed networks

The unipartite null model and the FOCS algorithm can be naturally extended to bipartite networks. The node set of a bipartite network is divided into two sides *U* and *V* such that each $u\in U$ can form edges only with nodes in *V*, and vice versa. Consider a candidate bipartite community $C=({C}_{U},{C}_{V})$, and an exterior node $u\in {C^{\prime} }_{U}:\,=U\backslash {C}_{U}$. In the bipartite null, analogously to the unipartite model, all outgoing edges from *C* and all edges between ${C^{\prime} }_{U}$ and ${C^{\prime} }_{V}\,:\,=V\backslash {C}_{V}$ are broken, and edge stubs coming from *u* are re-assigned without replacement. In this setting, the degree of *u* in *C*_*V*_ has the hypergeometric probability mass function

7 $$P({d}_{u}({C}_{V})=a)=\frac{(\begin{array}{c}{d}_{{C}_{V}}({C^{\prime} }_{U})\\ a\end{array})\,(\begin{array}{c}{d}_{{C^{\prime} }_{V}}({C^{\prime} }_{U})\\ {d}_{u}-a\end{array})}{(\begin{array}{c}{d}_{{C^{\prime} }_{U}}\\ {d}_{u}\end{array})}$$

**Figure 1:**
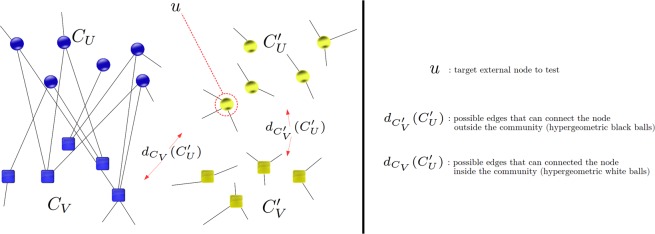
Illustration of bipartite null model for FOCS score. Circles and squares represent *U* nodes and *V* nodes, respectively. Blue nodes are the community to be scored. *u* is an external node for testing against the null model. The legend explains the edge counts’ role in the model.

The edge-breaking bipartite null model which produces the above distribution is illustrated in Fig. 1. Using (7) instead of (1), the rest of the FOCS approach follows unchanged, treating ${C^{\prime} }_{U}\cup {C^{\prime} }_{V}$ as the external nodes. As for directed networks, the use of FOCS depends on the type of community optimization, that is, whether in-degree, out-degree, or joint in-out-degree communities are being optimized. Each of these cases involves external node null distributions similar to that for undirected unipartite and bipartite cases, and can be used straightforwardly within the general iterative algorithm given above.

### Benchmarks and empirical studies

Here we describe the setup for and application of FOCS and existing methods to (1) communities from simulated null networks, (2) ground-truth communities from simulated community-laden networks, (3) standard real-world networks from the literature, and (4) a new actor-movie network introduced in this paper.

#### Simulation study

We perform two simulation experiments to examine the empirical Type-I error and power of the proposed and existing methodologies. In all experiments, the QS-Test and B-Score methods were run with default parameter settings (as presented in the associated papers and code manuals), and FOCS was run with $p=0.25$. The first simulation experiment involves “null” networks generated by the configuration model, which is a standard model for networks *without* communities^17^. Each network had 100 nodes, and the degree distribution was generated by a power law with exponent −2 on the range $[10,50]$. The total number of simulation repetitions was 1,000. At each repetition, the Louvain community detection algorithm was run^18^, and a community for scoring was chosen uniformly at random from the set of non-trivial communities (size > 2).

The second simulation experiment involves community-laden networks generated by the LFR model^19^, which will help assess the relative detection powers of the methods. The central parameter of this model is $\mu \in [0,1]$, which controls the average proportion of out-edges of each community. If *μ* is 1, all edges from each node point outside the node’s community, and if *μ* is 0, all communities are externally disconnected. Other parameters of the model control the distribution of community sizes and the degree distribution. In this experiment, four LFR network settings are tested: “small” networks with $n=1,000$ vs. “large” networks with $n=5,000$, and “small” communities with sizes in $[10,50]$ vs. “large” communities with sizes in $[20,100]$. Note that all these networks are tiny by today’s industry standard, but that QS-Test and B-Score are prohibitively slow on networks beyond this order of magnitude. In each setting, five LFR networks were simulated at each *μ* on an even grid, and the average significance scores for each method were computed across the ground-truth communities from all five repetitions.

#### Real data studies

We test FOCS, B-Score, and QS-Test on real-world network data sets commonly used in the network science literature. The data sets used were obtained from the open-access data repository KONECT^20^ and through links provided at Dr. Mark Newman’s website (http://www-personal.umich.edu/~mejn/netdata/), and were chosen so that these results could be compared to those from^11^. The data sets are listed, with references and numerical properties, in Table 1. To compare the FOCS, QS-Test, and B-Score methods on a particular data set, first the Louvain algorithm was run on the network 50 times, with different randomized initializations. Each method scored every Louvain community from the partition with the highest modularity, and the proportion of significant communities (score ≤ 0.05) is shown in Table 1. We describe insights from this study in the Results section.

**Table 1:** Description of real-world benchmark datasets, and summary numbers: number of nodes (#Nodes), number of edges (#Edges), number of communities found by the Louvain algorithm (#Com), and proportion of communities found significant (score < 0.05) by each method (columns labelled by method).

Network name	Network Description	#Nodes	#Edges	#Com	FOCS	B-Score	QS-Test
zachary^22^	karate club social	34	78	4	0.250	0.500	0.500
dolphins^23^	dolphin interaction	62	158	4	0.000	0.250	1.000
lesmis^24^	Les Miserables character appearance	77	254	6	0.167	0.333	1.000
enron^25^	ENRON email send/receive	87273	1148071	378	0.889	0.958	0.423
netscience^26^	network science collaboration	1589	2742	177	0.819	0.887	0.525
polblogs^27^	political blog hyperlink	1490	19090	7	0.286	0.571	0.000
airports^20^	U.S. airport flight	7976	30501	23	0.957	0.739	0.522
propro^28^	yeast protein interaction	1870	2277	76	0.066	0.184	0.461
chess^20^	chess game	7301	65052	36	0.722	0.583	0.361
astro-ph^29^	physical science collaboration	18771	198050	184	0.989	0.859	0.141
internet^20^	autonomous systems	34761	171402	39	0.051	0.179	0.692

Next, we tested FOCS on a network derived from a regularly updated IMDB database (https://datasets.imdbws.com). To display FOCS’s handling of diverse network types, we constructed a bipartite actor-movie network from the database. We used a snapshot of the database downloaded in November of 2018. We included as “actors” writers and directors, additionally. In the network, an actor and a movie share an edge if and only if the actor played a role in the movie. No actor-actor or movie-movie pairs share edges. The movie set was restricted to those released in the US with more than 100 ratings on IMDB, and the actor set was restricted to those with at least one movie from this set. The resulting network had 37,611 movies, 151,571 actors, and 362,850 edges. The existing community scoring methods discussed in this paper, QS-Test and B-Score, were not included in this application, because they do not handle bipartite graphs. We describe the application of FOCS and the results in the next section.

## Results

### Simulation study: configuration model (null) networks

**Figure 2:**
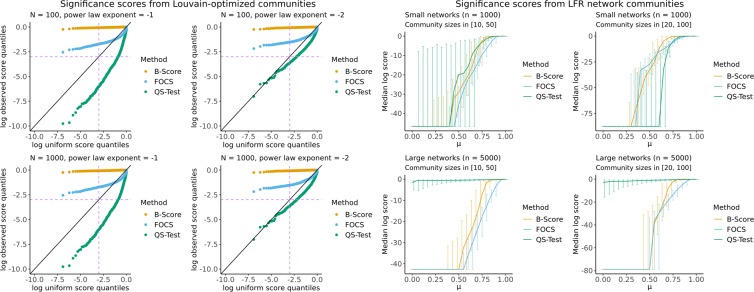
Left: Significance score log-distribution on configuration model networks. Purple dotted lines show significance cut-offs. Diagonal line is the expected Uniform distribution - conservative scores fall above this line, and anti-conservative scores below. Right: Results on the four tested LFR settings. Scores are on the log-scale, so that more negative values imply greater significance. Flat lines are across regions where raw scores went below machine precision.

As described in the previous section, we simulated 1,000 null networks with 100 nodes each, performed Louvain community detection, chose one resulting community uniformly at random, and scored the community with each method. In this way, we generated a null distribution of significance scores for both FOCS and existing methods. The left 2 × 2 plot in Fig. 2 shows the log-scale distribution of significance scores from the three methods, plotted against the grid of uniform quantiles that would be expected in a perfectly null distribution of scores. Purple dotted lines show the standard 0.05 significance cutoff under the log transformation. Therefore, the bottom-right quadrant formed by the purple dotted lines is the region in which observed scores would indicate significance but uniform-generated scores would not. The top-left quadrant is vice-versa. The figure suggests that the QS-Test is anti-conservative on null networks. In other words, applying the QS-Test with a significance cut-off of $\alpha =0.05$ to a given community will yield a probability of false positive greater than *α*. An explanation for this behavior is not obvious, as the method is performing simulations directly from a null model. The error may be due to poor interaction of the quality function’s kernel density estimator with the null model.

In contrast, the B-Score method is conservative for all values of *α*. The proposed FOCS method is conservative for $\alpha \le 0.5$, but slightly anti-conservative for larger *α*, which does not affect practical use as having false-positive rates greater than 50% is rarely acceptable protocol. Thus, we can see the trade-off effect of the FOCS null model’s relaxation of the perfect community optimization assumed by the B-Score method. FOCS is conservative for practical values of *α*, while less conservative overall than B-Score, which allows FOCS greater power in the presence of communities (as described in the next section).

### Simulation study: LFR networks

As described in the “Methods” section, our second simulation study involved simulating networks with planted communities of various strengths, and computing the median significance score from each method at each level of community strength. These median curves are displayed in the right 2 × 2 plot in Fig. 2, along with the 5% and 95% percentiles across repetitions. The *y*-axis corresponds to significance score, plotted on the log scale to amplify differences in extreme significance regions. On this scale, more negative values imply greater significance. The *x*-axis corresponds to the community strength (higher values imply more outward-linking and hence weaker communities). The right-hand plot in Fig. 2 shows that the detection power of the methods vary with both network size and community size. On small networks with small communities, FOCS is the dominant method. On small networks with large communities, FOCS is comparable to B-score, while QS-Test outperforms both these approaches. On large networks, FOCS is the dominant method, and surprisingly, QS-Test loses much of its detection power.

### Empirical study: standard real-world network benchmarks

Here we present the results of the proposed and existing methods on standard real-world networks from the literature. Table 1 shows information about the data sets, and the detection rates (significance score < 0.05) of each method.

#### Detection rates on real data

Two patterns from the detection rates in Table 1 reflect the LFR network simulation study. First, FOCS detection rates are more correlated with those from B-Score than those from QS-Test. Second, QS-Test detection rates are much lower on large networks, with the exception of the internet data set. Based on the simulation study, this exception may be due to the fact that the network had relatively larger communities. Note that in this study, higher detection rates does not necessarily suggest better performance, as not all communities in real networks are indicative of real-world ground truth - indeed, this is the reason optimized-community inference methods are useful. For instance, the FOCS method declared two communities significant on the political blogs data set, whereas B-Score declared four. However, the two communities FOCS found significant were the large communities corresponding to, respectively, liberal and conservative sentiments. Other smaller, less-focused communities were ignored, which is a reasonable result.

#### Stability and runtime

**Figure 3:**
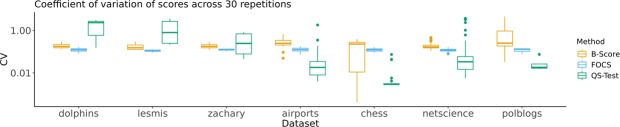
Boxplots of score coefficient of variations across communities, by method and dataset.

On some representative real data sets, each method’s significance score computation was repeated 30 times with different seeds, for the purposes of measuring (i) numerical stability and (ii) runtime. The largest data sets were not included in this study, as the runtimes for QS-Test and B-Score on these data sets were prohibitively long. Numerical stability was measured because each method has randomized steps in its algorithm. The metric used to measure stability on a fixed community and network is the coefficient of variation of the significance score (standard deviation score divided by mean score), across multiple runs of the algorithm. A low CV score implies that the randomized parts of the method being tested did not drastically affect the significance scores, on the particular community. Figure 3 shows boxplots of CV scores on a logarithmic scale, within each data set, across communities. The results show that each method had dominant numerical stability in some data set. However, interestingly, the FOCS CV metrics were by far the most consistent, which suggests that, in contrast to other methods, the expected numerical stability of FOCS scores does not depend on the particular community nor the particular data set, which is desirable.

The stability and runtime analyses were performed on 2.20 GHz Intel(R) Xeon(R) CPU E7-8890 processors, and the QS-Test computations were distributed across 24 cores, using parallelization options provided with the authors’ package (see https://github.com/skojaku/qstest). Computations for B-Score and FOCS methods were not parallelized. Table 2 gives the mean and standard deviation of runtimes of each method, over the computation repetitions. Note that each runtime (out of thirty runtimes) is the sum of the runtimes from each individual community. On all data sets, FOCS achieved the lowest average runtime compared with the other methods, often by one or two orders of magnitude.

**Table 2:** Average runtime in seconds of methods across 30 repetitions.

Method	dolphins	lesmis	zachary	airports	chess	netscience	polblogs
B-Score	0.35 ± 0.05	0.41 ± 0.00	0.13 ± 0.00	166.85 ± 4.25	1228.03 ± 70.62	4.92 ± 0.01	160.22 ± 0.72
FOCS	**0**.**16** ± 0.01	**0**.**16** ± 0.01	**0**.**16** ± 0.03	**1**.**40** ± 0.06	**5**.**37** ± 0.24	**0**.**53** ± 0.04	**0**.**88** ± 0.07
QS-Test	1.14 ± 0.05	1.37 ± 0.04	0.84 ± 0.01	74.66 ± 0.89	356.63 ± 3.22	28.17 ± 0.52	55.45 ± 0.75

QS-Test computations were distributed across 24 cores.

### Empirical study: Internet Movie Database (IMDB) network application

As described in the Methods section, we constructed a bipartite IMDB actor-movie network comprised of ~38 K movies and ~152 K actors. Here we discuss the results of FOCS scoring on communities from this network. Note that the published version of the existing methods discussed in this paper cannot handle bipartite networks, so we did not apply those methods to this data. To find optimized communities in the network, a single stage of the Louvain algorithm was performed until convergence on a local maximum of Barber’s bipartite modularity^21^. This produced 25,830 communities in the IMDB network with median size 6, and maximum size 16,467. A manual review of these communities would be cumbersome - this is where a method like FOCS becomes useful. Each community was scored with FOCS and ranked by decreasing FOCS score. Furthermore, ${g}_{P}(u,C)$ scores for each actor (see Section below Eq. 1), which indicate connectivity to the community, were computed and used to filter actors for display purposes.

The three highly-ranked communities are shown in Table 3, with all movies from each community listed, as well as the top six actors from each community by ${g}_{P}(u,C)$ score. Each filtered actor/director set included the major writers and lead actors and actresses from each film, showing the sensitivity of the node-wise *g*_*P*_ score to real-world signal. Note that “Daisy”, listed in the top community actor set, was the actual name of the dog featured in most of the Blondie titles. “Go West, Young Lady” is a film directed by Frank R. Strayer, director of the Blondie films, and stars Penny Singleton and other members of the Blondie movies, which explains its inclusion in the community. We also point out that the Blondie series, in fact, had the highest FOCS significance score among the discovered communities. This is due to the fact that film, as an entertainment genre, was relatively new at the time of the Blondie movies. Therefore, the participating actors and writers had few other projects, making the Blondie-movie community inordinately exclusive and concentrated.

**Table 3:** Top-ranked IMDB bipartite communities with well-known titles, ordered by FOCS score.

Size	FOCS	Movie Set	Actor Set
44	5 · 10^−16^	{24 out of 28 original Blondie titles}	Penny Singleton 4 · 10^−98^
		Go West, Young Lady	Larry Simms 2 · 10^−94^
			Chic Young 2 · 10^−94^
			Arthur Lake 7 · 10^−92^
			Daisy 2 · 10^−57^
			Frank R. Strayer 8 · 10^−48^
18	7 · 10^−11^	Harry Potter and the:	J. K. Rowling 1 · 10^−30^
		- Sorceror’s Stone	Rupert Grint 1 · 10^−30^
		- Prisoner of Azkaban	David Heyman 3 · 10^−28^
		- Goblet of Fire	Daniel Radcliffe 7 · 10^−27^
		- Order of the Phoenix	Steve Kloves 4 · 10^−24^
		- Half-Blood Prince	Emma Watson 8 · 10^−24^
		- Deathly Hallows pt 1	
		- Deathly Hallows pt 2	
12	2 · 10^−9^	Twilight	Melissa Rosenberg 6 · 10^−21^
		The Twilight Saga:	Stephenie Meyer 6 · 10^−21^
		- Eclipse	Robert Pattinson 8 · 10^−19^
		- Breaking Dawn pt 1	Kristen Stewart 3 · 10^−17^
		- Breaking Dawn pt 2	Taylor Lautner 1 · 10^−15^
		- New Moon	Karen Rosenfelt 3 · 10^−15^

These communities, and other highly-ranked communities not shown in the table, showed clear correspondence with stand-out actor/director collaborations. They featured well-known movie series or collections, paired with their directors, lead writers, and lead actors, and *only* those actors/directors/films that were linked to the series. Since the null model used by FOCS involves global re-assignment of edge stubs, it makes sense that focused, persistent activity by groups of actors across related films would receive the lowest significance scores. Communities with large FOCS scores exhibited much less internal coherence - from a manual inspection, many communities contained pairs of unrelated movies with few actor overlaps. This illustrates the fact that not every optimized community is meaningful in practice. It is a particular feature of a method like FOCS to be able to distinguish between these communities and important, strongly-connected communities that correspond to true underlying dynamics of a system.

**Figure 4:**
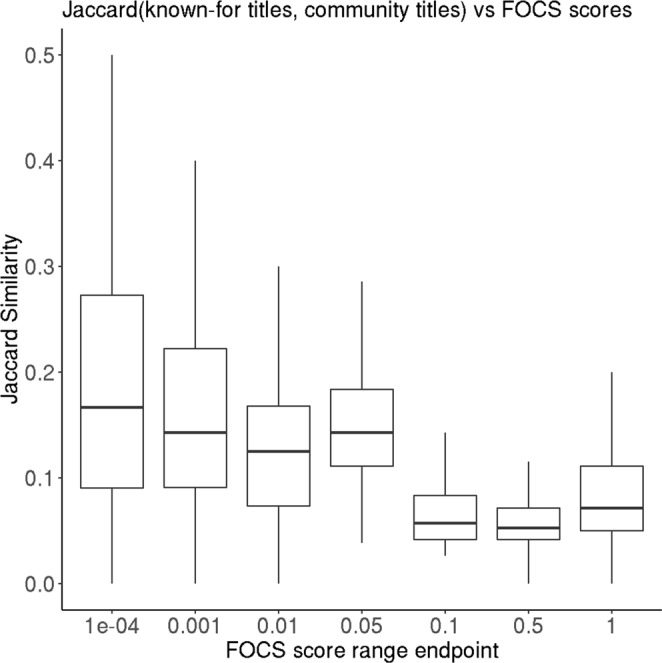
Distribution of jaccard similarities between cluster movie sets and actor “known-for” movie sets, across clusters, within ranges of FOCS scores. *x*-axis labels display the upper endpoint of the range, which extends back to the previous (left) upper endpoint. The lowest range extends to zero.

To quantitatively assess ground-truth quality of each community, for each actor, we determined the set of movies they are “known for” according to the IMDB metadata. Then, we compared these sets with the communities to which each actor belonged. Explicitly, we computed the jaccard similarity between each community’s movie set and, for the corresponding actor set, the union of the “known-for” sets. This metric is a proxy for the coherence of the community with respect to established cinema trends. The jaccard-based coherency metric correlated highly with FOCS significance scores (r = 0.1536, p = 9 × 10^−137^). Figure 4 shows a breakdown of jaccard scores by FOCS score range, showing a general upward trend as FOCS scores become lower on a quasi-logarithmic scale. This association shows that the manually identified patterns discussed above hold in general, and that in this application, the community ranking provided by FOCS aligned with meaningful real-world signal.

## Discussion

This paper introduces new models and tests for optimized communities in networks, and presents FOCS, an algorithm for significance scoring that has multiple performance benefits over existing approaches. FOCS uses a core scoring approach that relies on the fact that communities are rarely optimized perfectly, and therefore weakly connected nodes in communities distribute edges approximately according to a random graph null model. Because of this, FOCS has a simplicity that previous methods lack, making it more scalable, more numerically stable, and more easily generalizable. Despite its simplicity and speed, FOCS performs ahead of or comparably to preceding methods in terms of reduced tendency for false positives, and reduced significance scores on true communities. On a large-scale bipartite movie-actor network derived from IMDB data, the highest FOCS-ranked Louvain communities were those with highly related movie sets sharing continuous involvement from a dedicated cast and crew. This suggests that FOCS can be useful in detecting communities exhibiting anomalous, persistent involvement from its members.

The FOCS method’s main limitation is that the underlying null model is based on arguably plausible yet non-rigorous ideas about the distribution of nodes in communities that have been locally-optimized by partition-based community detection techniques. For these reasons, FOCS is not an exact statistical test, and its results should be reported with these caveats. It should be noted that existing methods also rely on approximations, which is often necessary when dealing with the intractable distributions of certain statistics under many graph models. Secondly, our null network model simulations showed that FOCS may be too conservative on useful ranges of the significance level. This means that there may be headroom to improve FOCS by making it less conservative and more powerful, which is an area for future research.

Despite these limitations, the FOCS method appears to improve greatly on the existing options for scoring the significance of individual communities. Given its scalability and straightforward implementation, it can be readily used in real-time anomaly detection, machine learning pipelines, and scientific studies. A basic implementation of the FOCS method used in experiments discussed in this paper can be found at https://github.com/google/fast-optimized-community-significance, and the pipeline of experiments can be reproduced with code at https://jpalowitch.github.io/focs_experiments/.
